# The impact of the COVID-19 pandemic on pregnancy, birth and sexual & reproductive health and rights: Perspectives from Germany and Somalia

**DOI:** 10.7189/jogh.11.03085

**Published:** 2021-09-04

**Authors:** Marrium Habib, Sabine Ludwig, Ute Lange, Clarissa Prazeres da Costa

**Affiliations:** 1 Center for Global Health, Technical University of Munich (TUM), Munich, Germany; 2 Department of Public Health Services Research, Bochum University of Applied Health Sciences, Bochum, Germany; 3 Center for Global Health, Charité – Universitätsmedizin Berlin, Berlin, Germany; 4 Department of Applied Health Sciences, Bochum University of Applied Health Sciences, Bochum, Germany

Past public health emergencies have shown that the impact of an epidemic on pregnancy, birth and SRHR, often goes unrecognized, because the effects are often not the ‘direct’ result of the infection but the ‘indirect’ consequences driven by supply and demand factors that disrupt the delivery of and access to essential health services [1]. Balancing the direct response to an acute pandemic situation with the continued delivery of other health services has been a universal dilemma for policymakers. The 2014-16 West Africa Ebola outbreak exemplifies the harmful impact of a public health emergency in the absence of focused national responses to protect the gains made in sexual and reproductive health (SRHR), low- and middle-income countries (LMICs), over the past several decades [2]. During the outbreak, fear of contagion resulted in fewer women attending health clinics [3]; which led to a 75% increase in maternal mortality in three affected countries [3]; and resulted in sharp declines in family planning visits and contraceptive uptake in Guinea, Liberia and Sierra Leone [4,5]. In Guinea, the proportion of antenatal care visits and institutional deliveries had not recovered to pre-epidemic levels even after six months after the epidemic, suggesting a sustained effect on the country’s already inadequate level of care [5]. In the present situation, similar factors at play during successive COVID-19 outbreaks could exacerbate women and girls’ health conditions, especially in an LMIC such as Somalia, which has some of the worst health indicators in the world following decades of conflict, natural disasters and disease outbreaks [6,7]. How then should decision makers evaluate different response options to effectively respond to COVID-19, while not aggravating all-cause morbidity and mortality from diverting care for other conditions? These complex questions merit nuanced considerations across both LMIC and HIC contexts. Hence, the WGH organized an online webinar via Zoom on 14th July 2020 to discuss how pregnancy, birth and SRHR are affected during the COVID-19 pandemic, within the German and Somalian health contexts. The WGH as a global movement that brings together all genders and backgrounds to achieve gender equality in global health leadership [8]. To frame the 90-minute discussion, two obstetricians and two midwives each from Germany and Somalia were invited to draw upon their experiences of service provision during the acute phase of the pandemic. This commentary can add to emerging evidence and can serve as a guide to policymakers and donors, to highlight the importance of preserving SRHR during the pandemic response and recovery periods.

## ROUTINE WORK IN COMMUNITY-BASED CARE

In Germany, the first four to six weeks of the lockdown led to the closure of the outpatient departments of the panelists’ facilities: routine antenatal and postnatal services (with the exception of monitoring of high-risk pregnancies) were partly disrupted. Labour wards had to be adjusted to physical distancing regulations leading to various logistical and regulatory challenges. Barring admittance to fathers in the labour ward increased the work for midwives and had negative psychosocial effects for the mothers. The Somalian panelists highlighted that the country was experiencing a shortage of midwives even prior to COVID-19, however in the pandemic they had additional tasks such as information, education and communication (IEC) and referrals for COVID-19, making their efforts to preserve routine service delivery even more challenging. Due to the restriction of ground transport during lockdown, lack of security in conflict-prone areas and fear of contagion in health facilities, many women preferred to access antenatal and postnatal services at the homes of community midwives, putting them at risk. This is also catastrophic to the gains Somalia has made in reducing maternal mortality by scaling up life-saving institutional deliveries, attended by trained midwives and obstetricians. For both country-contexts, midwives engaged in community care undertook great personal risk due to the lack of adequate protective equipment and physical contact with patients. The midwives from Germany stressed that while virtual care can be helpful; in-person care is often the only advisable mode of provision of specific pregnancy, birth and SRHR services. Various technical guidance documents have highlighted the role of dedicated spaces for the management of COVID-19 patients to reduce exposure risks among the non-infected and help alleviate fears about seeking essential services during this crisis [9]. However, there is a neglect for other services required during this time. The UNFPA recommends antenatal and postnatal care facilities and/or mobile clinics to support the continuum of maternal and newborn care [10]. The Somalian midwife highlighted UNFPA-funded comprehensive emergency care facilities where cesarean sections can be performed along with other life-saving interventions. Within the context of the pandemic, any reorganization or enhancement of health care facilities needs to be accompanied by clear communication, so that people know where to access adequate services. In the context of pregnancy, birth and SRHR services, appropriate screening, triage, and infection prevention and control strategies are mandatory to reduce the chance of introducing COVID-19 in such complicated care settings [10].

**Figure:**
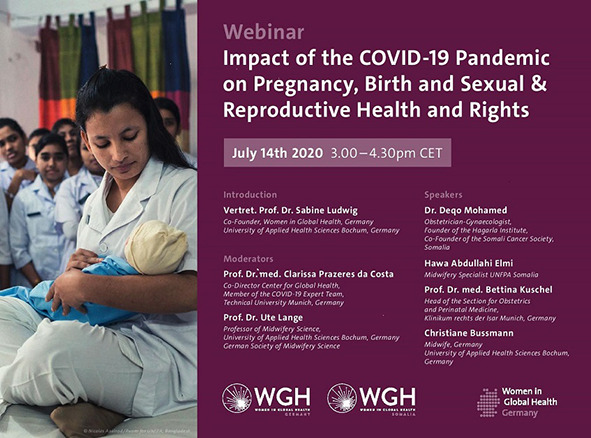
Photo: Webinar flyer (developed by Alfred Küng).

## SUPPLY-SIDE ISSUES FOR MEDICATION AND PERSONAL PROTECTIVE EQUIPMENT (PPE)

The panelists from both countries faced challenges with the supply of protective equipment and medication in the initial phases of the lockdown: German health care workers (HCWs) particularly faced a shortage of FFP2 masks; PPE provision for HCWs in the obstetrics and gynaecology was piecemeal and relied on private procurement in both countries. The panelists highlighted that Somalia still faces shortage of medication, with price hikes due to hoarding, and PPEs. Since the peak of COVID-19 hit Somalia later, a mask policy was already in place in health facilities, which was considered a positive development. Disruptions in the supply chain and parallel spikes in short-term demand, as COVID-19 hits new peaks globally, may present further challenges. A successful supply chain will likely require a combination of regulatory changes and international coordination [11]. In the LMIC context, support from development partners, and local collaboration with non-governmental organizations (NGOs) to facilitate access and delivery is particularly crucial [12]. Both country participants flagged the need to ensure protective equipment for community service providers to facilitate home visits and to scale up available resources to support virtual care and community-based monitoring. Technologies that enable home-based care present important opportunities to continue providing services outside clinics while also protecting HCWs from risk of infection [1].

## DIGITAL SOLUTIONS: MHEALTH, TELEMEDICINE AND PROFESSIONAL DEVELOPMENT ONLINE

The initial weeks of social distancing highlighted the significance of exploiting digital solutions for continuation of routine life. The panelists from Germany reported a positive uptake of webinars: emerging data about management strategies for COVID-19 were shared in real-time and international collaboration was facilitated. Midwifery colleagues reported positive experiences with virtual postnatal follow-ups, reporting good uptake by new mothers and more mother reached in the same time as before. However, data protection concerns remained a constraint to scaling up efforts to reach patients and colleagues through platforms such as Zoom and WhatsApp. The Somalian panelists flagged three significant outcomes: their understanding of COVID-19 was improved due to daily webinars sharing data emerging in HICs; the national call-center provided initial screening, triage, advice and referrals enabling unprecedented access; enthusiastic uptake for the use of platforms such as WhatsApp for postnatal follow-ups, SRHR services and general advice about COVID-19 for younger Somalian patients. However, it was noted that Somalian midwives were not prioritized for training in telemedicine and mHealth despite their significant role in service provision in the community. In both country contexts, exploring tools and platforms that can be leveraged across different programmatic areas for various aspects of care provision and IEC may offer strategic ways to remotely manage health needs during social distancing. Additionally, prioritizing the work of frontline workers (FLWs) in effectively disseminating IEC and tracking the real-time impact of COVID-19 on communities [13]. The Somalian national COVID-19 helpline provided access to a large proportion of the underserved: appropriately triaging and referring people to higher-level care. This strategy could be deployed in both country-contexts to promote antenatal care, help women plan for institutional deliveries and to connect them to family planning services [1]. While the use of these technologies must safeguard privacy and confidentiality, a state of technology inertia and regulatory limbo, should be avoided especially by HICs [14]. Germany can leverage its well-developed communication infrastructure and skilled human resources to maintain emergency response. In the longer term, investing in health data innovations and telemedicine, alongside facility and infrastructure upgrades, could be impactful contributions to emergency response situations.

## CONCLUSION

Panelists indicated that pregnancy, birth and SRHR are compromised in many settings during COVID-19, exacerbating many existing inequities and vulnerabilities. They highlighted the challenges faced and the gains made in the COVID-19 response in their routine work, in provision of essential services and in harnessing digital technologies to preserve the continuum of care. As the pandemic spreads globally, decision makers will require better data and evidence, well-adapted modelling tools, and nuanced guidelines. The WGH [8] has chapters in eleven HIC and LMICs including Somalia and Germany which could act as an important global platform for HCWs across different contexts to collaborate and strengthen global North-South partnerships eg, leveraging laboratory capacity and surveillance systems to support COVID-19 diagnostics and exchange experience and knowledge.
